# Population Pharmacokinetics Analysis To Inform Efavirenz Dosing Recommendations in Pediatric HIV Patients Aged 3 Months to 3 Years

**DOI:** 10.1128/AAC.02678-15

**Published:** 2016-05-23

**Authors:** Man Luo, Sunny Chapel, Heather Sevinsky, Ishani Savant, Brenda Cirincione, Richard Bertz, Amit Roy

**Affiliations:** a Bristol-Myers Squibb, Princeton, New Jersey, USA; b Ann Arbor Pharmacometrics Group, Ann Arbor, Michigan, USA

## Abstract

Efavirenz (EFV) is a nonnucleoside reverse transcriptase inhibitor approved worldwide for the treatment of HIV in adults and children over 3 years of age or weighing over 10 kg. Only recently EFV was approved in children over 3 months and weighing at least 3.5 kg in the United States and the European Union. The objective of this analysis was to support the selection of an appropriate dose for this younger pediatric population and to explore the impact of CYP2B6 genetic polymorphisms on EFV systemic exposures. A population pharmacokinetic (PPK) model was developed using data from three studies in HIV-1-infected pediatric subjects (*n* = 168) and one study in healthy adults (*n* = 24). The EFV concentration-time profile was best described by a two-compartment model with first-order absorption and elimination. Body weight was identified as a significant predictor of efavirenz apparent clearance (CL), oral central volume of distribution (*V_C_*), and absorption rate constant (*K_a_*). The typical values of efavirenz apparent CL, *V_C_*, oral peripheral volume of distribution (*V_P_*), and *K_a_* for a reference pediatric patient were 4.8 liters/h (4.5 to 5.1 liters/h), 84.9 liters (76.8 to 93.0 liters), 287 liters (252.6 to 321.4 liters), and 0.414 h^−1^ (0.375 to 0.453 h^−1^), respectively. The final model was used to simulate steady-state efavirenz concentrations in pediatric patients weighing <10 kg to identify EFV doses that produce comparable exposure to adult and pediatric patients weighing ≥10 kg. Results suggest that administration of EFV doses of 100 mg once daily (QD) to children weighing ≥3.5 to <5 kg, 150 mg QD to children weighing ≥5 to <7.5 kg, and 200 mg QD to children weighing ≥7.5 to <10 kg produce exposures within the target range. Further evaluation of the impact of CYP2B6 polymorphisms on EFV PK showed that the identification of CYP2B6 genetic status is not predictive of EFV exposure and thus not informative to guide pediatric dosing regimens.

## INTRODUCTION

Efavirenz (EFV) is a nonnucleoside reverse transcriptase inhibitor approved for the treatment of HIV-1 infection in adults and pediatric patients (1–3). EFV is principally metabolized by the cytochrome P450 (CYP450) system, and *in vitro* studies suggest that CYP2B6 is the major isozyme responsible for this process (4). EFV steady-state plasma concentrations are reached in 6 to 10 days (3). EFV has been shown to induce CYP450 enzymes, resulting in the increase of its own metabolism (autoinduction) (5). Long-term EFV autoinduction has been found to cause high interindividual (but low intraindividual) variability in the plasma pharmacokinetics (PK) (6). CYP2B6 polymorphisms also play a large role in interindividual variability. The 516G → T polymorphism of CYP2B6 occurs in 3 to 6% of Caucasians and 16 to 20% of African-Americans and has been associated with elevated systemic exposure and reduced clearance of EFV (7, 8). Additional CYP2B6 polymorphisms (e.g., 262K → R and 172Q → H) have also been reported which may also contribute to the variability in enzyme function, with the greatest impact observed in homozygous mutations (9).

Although EFV was first approved in the United States, the European Union, and other countries in the late 1990s for children 3 years of age and above and weighing more than 10 kg (1–3), EFV was only approved for children less than 3 years of age and weighing less than 10 kg in the United States in 2013 and in the European Union in 2015 (10, 11). One reason for the time lag between the initial approval of EFV and the approval for expanded pediatric patient populations is the challenge of conducting pediatric clinical trials. In addition to the unique ethical and operational hurdles of conducting pediatric studies, the main clinical pharmacology challenge is that pediatric patients represent a heterogenous patient population, which includes a wide range of physical characteristics (such as age, body weight, and maturation status) that may have an impact on the PK of the investigational drugs. Thus, it is critical to characterize the influence of the intrinsic and extrinsic factors of the pediatric population on the pharmacokinetic disposition of the investigational medicine to ensure appropriate dosing regimens. Extrapolation of adult PK and efficacy data has been shown to increase the efficiency of pediatric drug development, and a decision tree to guide pediatric development programs has been proposed by the FDA (12). PK-based extrapolation has been widely used to recommend pediatric doses of anti-HIV agents, given the similarities in HIV disease, and efficacy exposure-response relationships in children and adults (13; see also supplemental Table 7 in reference 12).

This article presents pharmacokinetic analyses that were performed to support the approval of EFV in the United States and the European Union for pediatric patients aged 3 months or older and weighing between 3.5 and 40 kg and to establish dose recommendations for these patients. A population pharmacokinetic (PPK) model that characterized the PK of EFV over a wide range of ages and body weights was used to identify appropriate doses of EFV that achieve the target adult exposure. Additionally, a pharmacogenomics assessment of the impact of CYP450 single nucleotide polymorphisms (SNPs) on EFV PK in pediatric patients was also performed, given its reported importance to the PK of EFV in adults (7, 8).

The analysis presented herein provided critical information for the approval of EFV in the United States and the European Union for pediatric patients aged 3 months or older and weighing between 3.5 and 40 kg. The newly approved dosing recommendations allow the use of a potent, well-tolerated, and simple (once daily [QD]) treatment regimen in HIV-1-infected pediatric patients less than 3 years of age.

## MATERIALS AND METHODS

### Study data.

Data from four clinical studies (three pediatric and one adult) were included in the analysis (Table 1). The pediatric studies (PACTG382, PACTG1021, and AI266922) assessed the safety and efficacy of EFV in pediatric patients and enabled the characterization of the PK following administration of EFV as an oral solution, capsule, capsule sprinkle, or tablet formulation. All PK samples were collected after EFV steady state was reached. The study in adult healthy volunteers (AI266059) was conducted to evaluate the relative bioavailability between intact capsule and capsule sprinkle formulations. The relative impact of the type of food vehicles mixed with capsule sprinkles was also investigated. All available PK data from these studies were included in the analysis.

**TABLE 1:** Summary of clinical studies used for PPK modeling

Study no.	Population	Study design	No. of subjects and treatment regimen (reference)
PACGT382	Pediatric patients, 3 to 16 yr	Phase I/II open-label, multicenter study	102 subjects dosed with EFV as an oral solution, capsule, or tablet formulation for up to 208 weeks, according to a dosing algorithm designed to achieve a target AUC_ss_, with a maximum daily dose of 1,000 mg (24)
PACTG1021	Pediatric patients, 3 mo to 21 yr	Phase I/II open-label study	43 subjects dosed with EFV as an oral solution, capsule, capsule sprinkle, or tablet formulation for up to 192 weeks; doses of EFV ranged from 360 mg to 720 mg depending on body wt (25)
AI266922	Pediatric patients, 3 mo to 6 yr	Phase II open-label, multicenter study	37 subjects dosed with EFV as an oral solution or capsule sprinkle formulation for up to 48 weeks; doses of EFV ranged from 300 mg to 720 mg depending on body wt
AI266059	Healthy adults	Open-label, randomized, 3-period, 3-treatment, crossover study	24 subjects dosed with EFV capsules or capsule sprinkle formulation under fasted conditions or mixed with various food vehicles (15)

There were three main sections to the analysis described herein. The initial model development was performed on all available data at that time (model development data set) to facilitate timely results to inform decision making. The model development data set comprised 4,125 EFV concentration values from 185 patients; 2,893 concentration values (the majority of which were sparsely sampled) were from 161 pediatric patients (studies PACTG382, PACTG1021, and AI266922), and 1,232 concentration values (intensively sampled) were from 24 healthy adults in study AI266059. After model development and covariate selection, the final PPK model from the development data set was then reestimated using the updated data set, which was comprised of the model development data set and additional newly available data. The updated PPK analysis data set included a total of 4,521 concentration values from 192 patients. The population for the updated data set comprised 168 pediatric patients (studies PACTG382, PACTG1021, and AI266922) and 24 healthy adults (study AI266059). Pediatric patients were 3 months to 21 years of age at the initiation of treatment and weighed 3.3 to 117 kg at the time of dosing (Table 2). The observations included 3,289 concentration values from pediatric patients (the majority of which were sparsely sampled) and 1,232 concentration values from adult patients (intensively sampled). The pediatric trials contributed 88% of subjects and 73% of observations in the updated data set. An analysis evaluating the impact of pharmacogenomics information was conducted as the final stage.

**TABLE 2:** Baseline characteristics of subjects (updated data set)

Parameter	Value for the parameter in:	
	Pediatric studies	Adult study
No. of participants^*a*^	168	24
Mean age (yr [min, max])^*b*^	6.73 (0.2, 24.7)	32.8 (20.0, 45.0)
Mean wt (kg [min, max])^*b*^	25.3 (3.3, 117)	80.6 (59.6, 98.1)
Sex (no. [%])		
Male	80 (47.6)	23 (95.8)
Female	88 (52.4)	1 (4.17)
Race (no. [%])		
White	56 (33.3)	12 (50)
Black/African-American	88 (52.4)	12 (50)
Other	24 (14.3)	0 (0)

a Values represent subjects with available PK data (93 for PACTG382, 41 for PACGT1021, and 34 for AI266922) and may be less than the number of treated subjects in the studies.

b Statistics on age and body weight were calculated based on the measurements taken at each clinic visit throughout the treatment for pediatric studies and at the baseline for the adult study. No Asian subjects were enrolled in any studies. Min, minimum; max, maximum.

### Population pharmacokinetic analysis.

The PK of EFV was characterized by a compartmental PPK model, using a nonlinear mixed-effects modeling approach. The PPK model was developed in three steps. First, a base model was developed to describe the PK of EFV without consideration of covariate effects. Second, a full covariate model was developed by incorporating the effect of prespecified covariate-parameter relationships. Last, the final model was chosen by retaining only the statistically significant covariate effects. The final model was evaluated using diagnostic plots and a prediction-corrected visual predictive check (pcVPC) (14). The base, full, and final models were developed with the model development data set. Subsequently, the final model parameters were reestimated with the updated data set, and additional model evaluation was performed to verify the adequacy of the final model to describe the updated data set.

The PPK model was developed using the first-order conditional estimation with interaction in NONMEM (version VI). All data processing was performed using SAS (version 8.2 or higher) and/or S-plus (version 7.0 or higher).

### Development of the base model.

Initially a base model was developed to describe the steady-state PK of EFV. A two-compartment model with first-order absorption and elimination was selected, specified in terms of absorption rate constant (*K_a_*), apparent oral clearance (CL), apparent oral central volume of distribution (*V_C_*), apparent oral peripheral volume of distribution (*V_P_*), and apparent intercompartmental clearance between the central and peripheral compartments (*Q*). The relative bioavailability (*F*_rel_) of the solution formulation was estimated relative to the capsule formulation, and the bioavailability of the capsule sprinkle formulation was assumed to be the same as that of capsules, as a study in adult subjects established that capsule sprinkles mixed with various food vehicles were bioequivalent to the capsule administered under fasted conditions with regard to the EFV area under the concentration-time curve at steady state (AUC_ss_) (15). The interindividual variability (IIV) in these PK parameters was assumed to be log-normally distributed in the population, except *F* (for which IIV was not estimated). The IIV in parameter *P* is given by *P_i_* = *P*_TV_ · exp (η_*P*,*i*_), where *P_i_* is the value of parameter *P* for the *i*th individual, *P*_TV_ is the typical value of parameter *P*, and η_*P*,*i*_ ∼ *N*(0, ω^2_*P*_^) is a normally distributed random variable with zero mean and variance ω^2_*P*_^, which represents random interindividual variability in *P*. For the interindividual random effects, several forms of the variance-covariance (Ω) matrix structure were explored. The residual variability was also assumed to follow a log-normal distribution, characterized by a log-transformed normal distribution with a zero mean and variance σ^2^.

EFV autoinduction was not incorporated into the model as PK sample collection in the pediatric studies occurred following approximately 2 weeks of daily dosing, by which time autoinduction is expected to have reached steady state (3).

### Development of the full model.

The full covariate model was developed by incorporating the effect of all prespecified covariate-parameter relationships, which were determined by clinical judgment and mechanistic plausibility. The covariate-parameter relationships assessed were as follows: the effects of age, weight, gender, race, prior antiretroviral therapy (PART; indicator of study PACTG1021), and concomitant protease inhibitor (PINT; nelfinavir or indinavir, indicator of study PACTG382) on CL; the effect of body weight on *V_C_*_;_ and the effects of age, weight, and formulation on *K_a_*. For continuous covariates (age and body weight), the relationship between the typical value of a parameter (*P*_TV_) and a continuous covariate (*R*) at each clinic visit was tested using the equation *P*_TV_ = P1(*R*/*R*_ref_)^*P2*^, where *P1* and *P2* are fixed-effect parameters and *R*_ref_ is a reference value of the covariate. Both age and weight were incorporated as time-varying covariates. For categorical covariates (sex, race, prior antiretroviral therapy, concomitant protease inhibitor, and formulation), the relationship between the typical value of a parameter *P*_TV_ and a categorical covariate (*R*) was tested using the equation *P*_TV_ = *P1* (*1 + R* · *P2*), where *P1* and *P2* are fixed-effect parameters. Covariates were included simultaneously using a full-model approach, followed by the procedure for Wald's approximation method (WAM) (16).

### Development of the final model.

The WAM procedure, which ranks all possible submodels (2^*k*^ submodels, where *k* is the number of covariates) derived from the presence or absence of the *k* covariate parameters in the full model, was used as a screening step to identify a subset of reduced PK models relative to the full model. The Bayesian information criterion (BIC) (17) generated during the WAM procedure was used to rank all possible models, and the top 15 ranked models were selected, which were then fit using NONMEM to calculate the actual BIC statistics. The final parsimonious model was selected based on the maximum NONMEM-based BIC among these top 15 ranked models. Backward elimination was performed to obtain the final model.

### Final-model evaluation.

The final model was evaluated using a prediction-corrected visual predictive check (pcVPC) (14). The pcVPC was performed with 1,000 sets of concentration values simulated from the final PPK model. The model was evaluated by comparing the median and 5th and 95th percentiles of the observed concentration-time profile of selected subgroups of subjects in the analysis data set, with the corresponding 90% prediction intervals (PIs). Summary statistics of the observed values at each nominal sampling time point were compared with simulated PIs by age and body weight groups.

### Model-based simulation to support pediatric dose recommendations.

Simulations using the final model were performed to determine weight-based dosing for patients weighing <10 kg; these simulations result in population mean exposures expected to be similar to the exposures achieved in adult and pediatric patients weighing ≥10 kg with the previously approved EFV treatment regimen. The recommended pediatric dose regimens were selected based on clinical judgment, regulatory requirements, and covariates determined to be clinically relevant in the final PK model (body weight).

Steady-state EFV concentration-time curves were simulated in pediatric patients (*n* = 100 per weight category) for selected dose regimens of the capsule and capsule sprinkle formulations. Subject demographics were sampled with replacement from the observed data set for simulation. The weight categories evaluated were 2.5 to 5, ≥5 to <7.5, and ≥7.5 to 10 kg. For each subject, EFV concentration values were simulated at nominal PK sampling times of 0, 1, 2, 4, 6, 8, 12, and 24 h postdose at steady state. For each of the simulated data sets, the mean individual EFV area under the concentration-time curve at steady state (AUC_ss_; 0 to 24 h) was calculated for each weight category. The distribution of the mean AUC_ss_ across 1,000 simulations was used to recommend the appropriate dose regimen for the corresponding weight category. The AUC_ss_ for this population was targeted to the range of the median to 2× median of the observed AUC_ss_ values in adults treated with 600 mg of EFV QD (190 to 380 μM · h) (3).

In addition to AUC_ss_ values, simulated maximum plasma concentrations (*C*_max_) and trough concentrations (*C*_min_) were evaluated. The parameters from children weighing ≥10 to <15 kg were used as reference values. *C*_max_ and *C*_min_ were deemed acceptable when the simulated values for patients weighing <10 kg were within the 80% to 125% range of the reference values (for *C*_max_, 5.2 to 8.2 μg/ml; for *C*_min_, 1.9 to 2.9 μg/ml) (3).

### Impact of CYP2B6 polymorphism on EFV PK.

Following completion of the final pediatric model evaluation and simulations, an *ad hoc* exploratory assessment of the impact of relevant CYP450 SNPs on EFV exposure was performed. The pharmacogenomic data were not included in the formal covariate analysis as these data were available only for subjects in studies AI266922 and PACTG382. Subjects who had wild-type homozygous alleles were designated 516GG, subjects who were heterozygous were designated 516GT, and subjects with mutations in both alleles were designated 516TT.

The effect of CYP2B6 polymorphism on EFV CL was assessed by two *ad hoc* analyses. In one of these analyses, the effect of the available data for the 516G → T allele on EFV CL was assessed directly by inclusion of the available data as an additional covariate in the final model. In the other analyses, the effect of polymorphisms on drug-metabolizing enzymes was assessed indirectly by a mixture modeling approach, whereby the CL in model-identified subpopulations was estimated.

In the analysis using the available polymorphism data as an additional covariate, pediatric patients who carried the wild-type CYP2B6 (516GG) were used in the modeling as the reference. The following expression was used to estimate the effect of the 516G → T polymorphism on CL: CL = CL_pop_(1 *+* θ*x* · 516GT)(1*+* θ*y* · 516TT)(1 *+* θ*z* · [516 missing]), where CL_pop_ is the population estimate of EFV CL for the final model in wild-type patients (516GG) after all selected covariate effects were incorporated, and θ*x*, θ*y*, or θ*z* is the fractional change in a parameter for a patient who is either a carrier of the 516GT (1, yes; 0, no) or 516TT (1, yes; 0, no) polymorphism or missing information (1, yes; 0, no) about the polymorphism, respectively. A term for missing values of the pharmacogenomic data was included to enable inclusion of all data in the estimation of structural model parameters.

In addition, a mixture model was developed by incorporating independent estimates of relative CL values for the three metabolizing subgroups (extensive, intermediate, and poor metabolizers) or two subgroups (extensive and poor metabolizers). Also, due to the physiologic differences associated with growth and development and the immaturity of enzyme systems and clearance mechanisms in children, the impact of different metabolizing subgroups on EFV PK may differ between children and adults. Therefore, both same-effect and separate-effect analyses of each subgroup on EFV PK for adult and pediatric patients were assessed. The proportion of adult and pediatric patients who belong to the three or two metabolizing subgroups was also estimated separately. The final mixture model was selected based on the maximum BIC for the model tested and corresponding diagnostic plots.

## RESULTS

### Population pharmacokinetic analysis. (i) Base-model development.

The steady-state PK of EFV was characterized by a two-compartment model with first-order absorption and elimination. The PK parameters for pediatrics and adults were estimated separately (equations as below). The absorption lag time (*T*_lag_) was necessary for adults but not for pediatric patients. Diagnostic plots with the initial base model indicated differences between EFV capsule sprinkle and oral solution. Diagnostic plots also indicated that the relative bioavailability of the oral solution was study dependent. Therefore, it was necessary to estimate the study-specific *F*_rel_ for the pediatric studies to adequately describe the data. The solution formulation also showed higher residual variability than the capsule sprinkle formulation. In the base model, bioavailability of the oral solution was 17%, 27%, and 69% lower than that of the capsule formulation in the PACTG382, PACTG1021, and AI266922 groups, respectively. The residual variability for the oral solution was higher than for the capsule formulation (67% versus 45%).

### (ii) Full-model development.

The full model was constructed with prespecified covariate effects, with separate covariate-parameter relationships for adult and pediatric subjects. The covariate effects on typical values of CL, *V_C_*, *K_a_*, and *F*_rel_ in pediatric (ped) subjects were described by the following: 

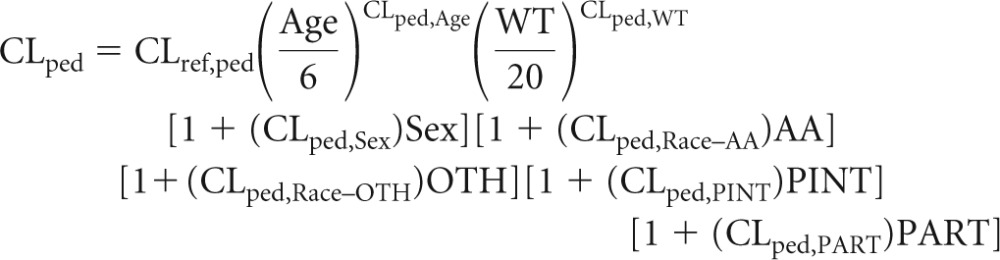

$$V_{C\text{ped}}={V_{C\;\text{ref},\text{ped}}\left(\frac{\text{WT}}{20}\right)}^{V_{\text{ped},\text{WT}}}$$

$$K_{a\;\text{ped}}=K_{a\;\;\text{ref}}(\frac{\text{Age}}{6}{)}^{K_{a\;\text{ped},\text{Age}}}\;(\frac{\text{WT}}{20}{)}^{K_{a\;\text{ped},\text{WT}}}$$

$$F_{\text{rel},\text{ped}}=1+(F_{\text{ped},\text{PACTG}382})(\text{SOLN})(\text{PACTG}382)+(F_{\text{ped},\text{PACTG}1021})(\text{SOLN})(\text{PACTG}1021)+(F_{\text{ped},\text{AI}266922})(\text{SOLN})(\text{AI}266922)$$

The covariate effects on typical values of CL, *V_C_*, *K_a_*, and *F*_rel_ in adult subjects were described by the following:

$$\text{C}{\text{L}}_{\text{adult}}=\text{C}{\text{L}}_{\text{ref},\text{adult}}$$

$$V_{C\;\text{adult}}=V_{C\;\text{ref},\text{adult}}$$

$$K_{a\;\text{adult}}=0(\text{if}\;\text{t}<T_{\text{lag}})\;\text{or}\;K_{a\;\text{ped}}\left(\text{otherwise}\right)$$

$$F_{1\;\text{adult}}=1$$

where Age, WT, Sex, PINT, and PART represent the age at each visit, body weight at each visit, sex (1, female; 0, male), concomitant protease inhibitor (1, yes; 0, no), and prior antiretroviral therapy (1, yes; 0, no), respectively. AA is 1 and OTH is 0 if the subjects are African American; AA is 0 and OTH is 0 if patients are either white or Asian; AA is 0 and OTH is 1 otherwise. SOLN is the indicator for solution formulation. PACTG382, PACTG1021, and AI266922 are the study indicators (1 if the subject is in the study or 0 otherwise). The reference (ref) values for age and body weight were approximate means of the observed values at dosing in pediatric studies.

The apparent intercompartment clearance between central and peripheral compartments (*Q*) and the apparent peripheral volume of distributions (*V_p_*) were kept the same for the adult and pediatric populations.

The parameter estimates for all fixed-effect and random-effect parameters for the full model using the model development data set are provided in Table 3.

**TABLE 3:** Parameter estimates of the full and final models

Effect type and parameter^*a*^	Estimate ± SE	
	Full model^*b*^	Final model^*c*^
Fixed effects		
CL_ref,ped_ (liters/h)	5.67 ± 1.15	4.8 ± 0.33
CL_ref,adult_ (liters/h)	3.66 ± 0.297	3.66 ± 0.294
CL_ped,WT_	0.673 ± 0.23	0.57 ± 0.107
CL_ped,Age_	−0.0201 ± 0.136	
CL_ped,Sex_	−0.0906 ± 0.0963	
CL_ped,Race-AA_	0.135 ± 0.14	
CL_ped,Race-OTH_	−0.0781 ± 0.128	
CL_ped,PINT_	−0.199 ± 0.165	
CL_ped,PART_	−0.458 ± 0.155	0.381 ± 0.401
*V_C_*_ref,ped_ (liters)	91.3 ± 8.86	84.9 ± 8.13
*V_C_*_ref,adult_ (liters)	186 ± 15	188 ± 14.9
*V_C_*_ped,WT_	1.39 ± 0.176	1.35 ± 0.152
*Q*_ref_ (liters/h)	5.47 ± 0.753	6.01 ± 0.839
*V_P_*_ref_ (liters)	286 ± 33.6	287 ± 34.4
*K_a_*_ref_ (h^−1^)	0.431 ± 0.05	0.414 ± 0.0387
*K_a_*_ped,WT_	0.817 ± 0.396	0.768 ± 0.0844
*K_a_*_ped,Age_	−0.115 ± 0.251	
*T*_lag_ (h)	0.62 ± 0.0383	0.633 ± 0.0357
*F*_rel_ for solution		
Study PACTG382	−0.355 ± 0.0896	−0.346 ± 0.0803
Study PACTG1021	−0.52 ± 0.0938	−0.0509 ± 0.344
Study AI266922	−0.753 ± 0.0532	−0.754 ± 0.0518
IIV random effects (variances)		
IIV_CL_ped_	0.371 ± 0.0650	0.602 ± 0.231
IIV_CL_adult_	0.158 ± 0.0312	0.158 ± 0.0312
IIV_*V_C_*_, ped_	0.244 ± 0.0678	0.234 ± 0.0651
IIV_*V_C_*_, adult_	0.132 ± 0.0453	0.132 ± 0.0435
IIV_*Q*	0.810 ± 0.173	0.695 ± 0.164
IIV_*V_P_*	0.296 ± 0.0874	0.296 ± 0.0877
IIV_*K_a_*	0.186 ± 0.0645	0.202 ± 0.0570
Residual error random effects (variances)		
Capsule, pediatric studies	0.432 ± 0.0287	0.461 ± 0.0286
Solution, pediatric studies	0.662 ± 0.0632	0.784 ± 0.101
Adult study	0.212 ± 0.00864	0.212 ± 0.00862

a Pediatric (ped) reference (ref) values of CL, *V_C_*, and *K_a_* are for a reference pediatric subject (male, body weight of 20 kg, aged 6 years, race of non-African-American and not other, no prior antiretroviral therapy and concomitant protease inhibitor). Covariate effects for these parameters are given with respect to the reference values. Adult reference values of parameters are for all adult subjects (as there were no adult-specific covariates). WT, weight; PINT, concomitant protease inhibitor; PART, prior antiretroviral therapy; *T*_lag_, absorption lag time; IIV, interindividual variability.

b These full-model parameter values were estimated based on the model development data set.

c These final-model parameter values were estimated based on the updated data set.

**FIG 1:**
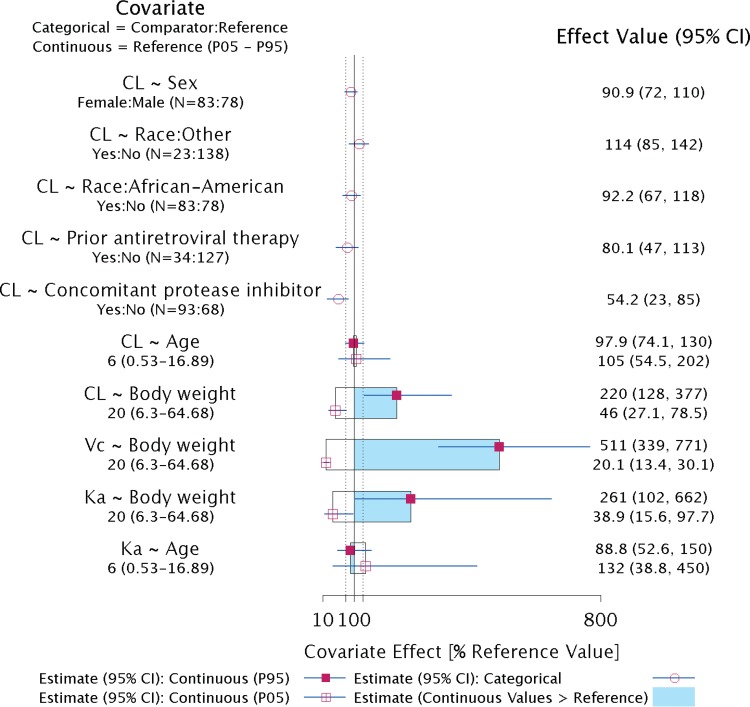
Covariate effects on pediatric PK parameters. The effects of covariates on efavirenz apparent CL, *V_C_*, and *K_a_* in pediatric subjects (estimated with the full model) are shown relative to the parameter values of a reference pediatric subject (male; body weight, 20 kg; age, 6 years; race, non-African-American and not other; no prior antiretroviral therapy; concomitant protease inhibitor). The dashed vertical lines represent 75% and 120% of the reference subject parameter value. P05, 5th percentile; P95, 95th percentile; CI, confidence interval.

Results of the full model indicated that body weight is the most influential covariate for CL, *V_C_*, and *K_a_* and that PART (indicator of PACTG1021) is a predictor of CL (Fig. 1). The effects of other covariates on CL, *V_C_*, and *K_a_* were within ±25% (Fig. 1) of the reference population, which indicates that they are unlikely to be clinically relevant.

### (iii) Final-model development.

Following the WAM and backward elimination procedures, the effects of age, sex, race, and PINT on CL were no longer statistically significant and so were removed from the model. Likewise, the effect of age on *K_a_* was no longer statistically significant and so was removed from the model. The final parsimonious model included the effects of weight and PART on CL, weight on *V_C_*, and weight on *K_a_*. After the final model was selected, the final-model parameter values were reestimated using the updated data set, and they are provided in Table 3. The final-model parameter values using the model development data set are provided in Table S1 in the supplemental material.

### (iv) Final-model evaluation.

**FIG 2:**
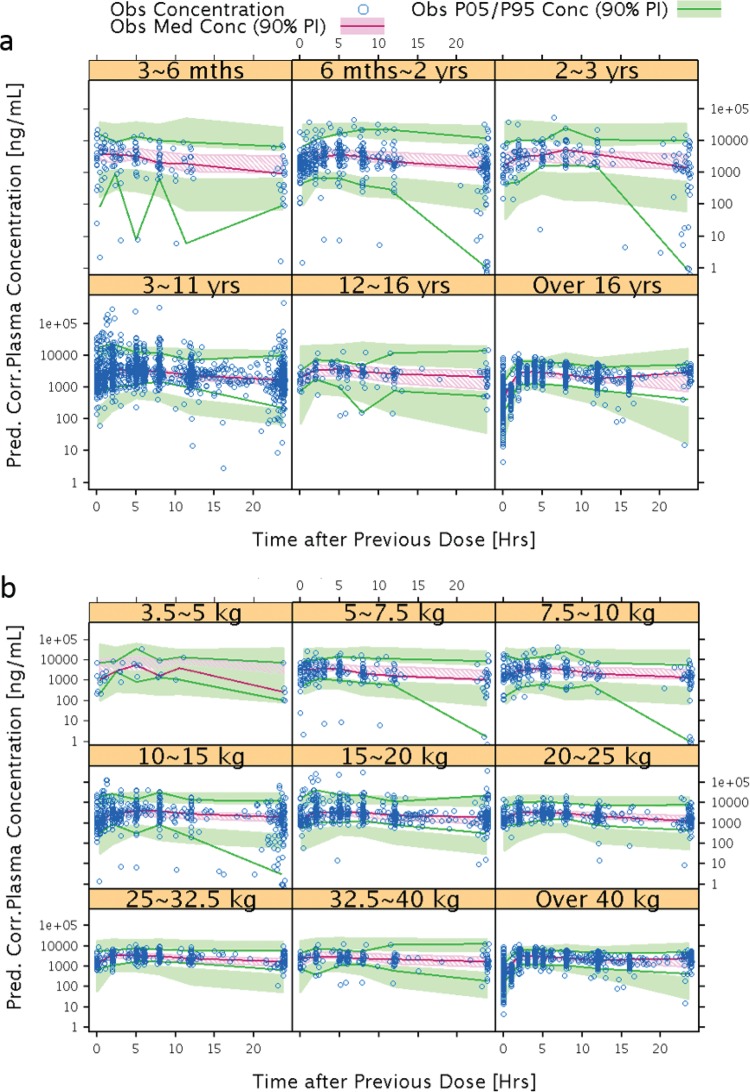
(a) pcVPC of observed plasma concentrations and 90% prediction intervals of simulated data for efavirenz by age group. (b) pcVPC of observed plasma concentrations and 90% prediction intervals of simulated data for efavirenz by weight group. mths, months.

The PPK model was evaluated using standard diagnostic plots and pcVPC plots with the updated data set. The diagnostic plots of model predictions versus observations, residuals versus model predictions, and residuals versus time showed that the model described the observed data adequately (data not shown). The pcVPC plots, which were further conducted, show the observed median and 5th and 95th percentiles of the EFV concentration profile within various time intervals, overlaid with the corresponding 90% prediction intervals from simulations for the pediatric and adult studies. These plots were stratified by six age groups in the data shown in Fig. 2a and by eight body weight groups in the data shown in Fig. 2b. Overall, the observed median and the 95th percentile of EFV concentrations generally fell within the 5th and 95th percentiles of the predictive distribution for the final model across time intervals and across age and weight groups (Fig. 2a and b). In the data shown in Fig. 2a, the 5th percentiles were less well described, especially in the 3- to 6-month age group. A potential reason that the observed 5th percentile was not well characterized by simulation may largely be due to the limited number of subjects (*n* = 10) in the 3- to 6-month age group. The pcVPC plots for other age groups (6 months to 2 years, 2 to 3 years, 3 to 11 years, 12 to 16 years, and >16 years) were well characterized. In the data shown in Fig. 2b, the lowest weight band (3.5 to 5 kg) was not as well characterized as the other eight weight bands, which was likely due to the small sample size (*n* = 5) in this group. The model provides reasonably adequate descriptions of the EFV concentration-time profile of pediatric patients in the remaining eight weight groups. In general, it was concluded that the final model provides adequate predictive performance of the central tendency of the EFV concentration-time profiles.

### Model-based simulations to inform EFV pediatric dosing.

**FIG 3:**
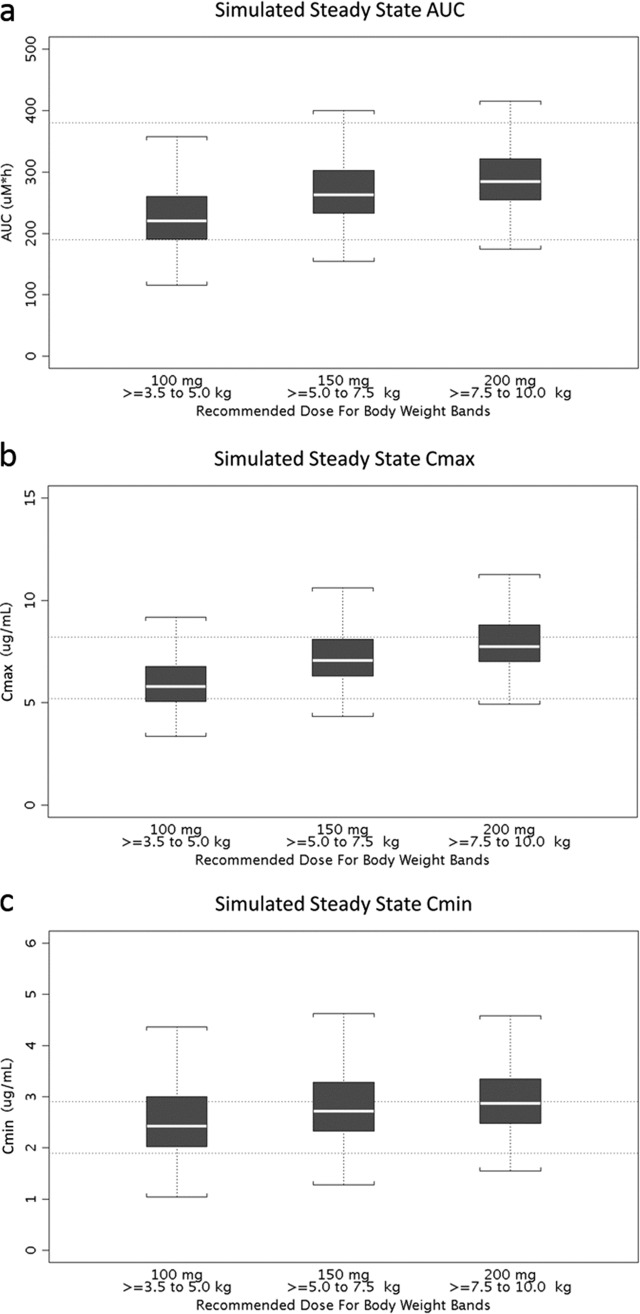
Target ranges based on adult data (dashed horizontal lines) and simulation results of the median (white lines) and 10th to 90th percentiles (whisker) of the mean individual EFV AUC_ss_ (0 to 24 h), *C*_max_, and *C*_min_ values.

EFV exposures were simulated for selected doses ranging from 100 to 200 mg, based upon doses selected for each weight group. Simulation results of the proposed pediatric dose regimens for EFV in patients weighing <10 kg are shown in Fig. 3. A weight band of 2.5 to <5 kg was originally simulated; however, the lowest observed body weight in the data set was 3.3 kg. Therefore, this lowest weight band was truncated to ≥3.5 to <5 kg. Using the original simulated data set for the weight band of 2.5 kg to <5 kg described above (1,000 simulations with 100 pediatric subjects per simulation), all simulated subjects with a body weight of <3.5 kg were excluded (*n* = 27 excluded). This resulted in 73 simulated subjects in each simulation (*n* = 1,000 simulations) in the weight band of 3.5 kg to <5 kg (a total of 73,000 virtual subjects in the weight band of 3.5 to <5 kg).

The simulation results suggest that EFV capsule sprinkle doses of 100, 150, and 200 mg QD for children who weigh ≥3.5 to <5 kg, ≥5 to <7.5 kg, and ≥7.5 to <10 kg, respectively, appear to produce EFV exposures similar to the target ranges. For the weight bands of ≥5 to <7.5 kg and ≥7.5 to <10 kg, the simulated mean AUC_ss_ values at 150 mg and 200 mg, respectively, were most similar to observed adult AUC_ss_ values (190 to 380 μM · h). *C*_max_ and *C*_min_ were deemed acceptable when simulated for patients weighing <10 kg as the values were within 80 to 125% of reference values (*C*_max_ and *C*_min_ were 5.2 to 8.2 and 1.9 to 2.9 μg/ml, respectively), which were the median of the mean values from children who weighed 10 to <15 kg across 1,000 simulations.

### Impact of CYP450 polymorphism on EFV PK.

The summary statistics for the key pharmacogenomics data are shown in Table 4. The genetic status information for CYP2B6 15631GT and 21563CT was available for a total of 102 pediatric subjects, and 12.7% of the subjects had homozygous mutations.

**TABLE 4:** Summary of key pharmacogenomics data from studies AI266922 and PACTG382

Covariate	No. (%) of patients
CYP2B6 15631GT	
Wild type	48 (47.1)
Heterogeneous mutation	41 (40.2)
Homozygous mutation	13 (12.7)
CYP2B6 21563CT	
Wild type	48 (47.1)
Heterogeneous mutation	41 (40.2)
Homozygous mutation	13 (12.7)

### (i) CYP2B6 polymorphism as a covariate on CL.

The effect of CYP2B6 single nucleotide polymorphisms (SNPs) on the systemic exposure of EFV was first assessed as a covariate on CL in the final EFV PPK model using CYP2B6 15631GT as the sole CYP2B6 genetic marker, as described above. The effect of CYP2B6 polymorphisms on EFV CL, as well as other model parameter estimates, is shown in Table 5. Pediatric patients who carried the wild-type CYP2B6 (516GG) were used as the reference. The model predicted an approximate 60% decrease for the mean estimates of EFV apparent oral clearance (CL) in pediatric subjects who were homozygous for the 15631GT substitution (15631TT) relative to that in pediatric subjects who did not carry the substitution on either allele (15631GG) and an approximate 25% decrease in pediatric subjects who carried the substitution on one allele (15631GT) relative to that in subjects who carried the wild type. These results were generally consistent with those reported by Haas et al. (7) for the effect of CYP2B6 polymorphisms in adults.

**TABLE 5:** Parameter estimates of the final model with CYP2B6 genetic information as a covariate

Effect type and parameter^*a*^	Estimate ± SE
Fixed effects	
CL_ref,ped_ (liters/h)	5.65 ± 0.522
CL_ref,adult_ (liters/h)	3.66 ± 0.293
CL_ped,WT_	0.593 ± 0.110
CL_ped, PART_	−0.0671 ± 0.342
CL_ped,CYP2B6-15631GT_	−0.244 ± 0.0832
CL_ped,CYP2B6-15631TT_	−0.613 ± 0.0837
CL_ped,CYP2B6-15631 missing_	0.239 ± 0.244
*V_C_*_ref,ped_ (liters)	85.5 ± 7.69
*V_C_*_ref,adult_ (liters)	188 ± 14.6
*V_C_*_ped,WT_	1.35 ± 0.157
*Q*_ref_ (liters/h)	6.05 ± 0.789
*V_P_*_ref_ (liters)	287 ± 25.3
*K_a_*_ref_ (h^−1^)	0.415 ± 0.0360
*K_a_*_ped,WT_	0.764 ± 0.0812
*T*_lag_ (h)	0.632 ± 0.0353
*F*_rel_ for solution	
Study PACTG 382	−0.324 ± 0.0852
Study PACTG 1021	−0.0644 ± 0.343
Study AI266922	−0.754 ± 0.0512
IIV random effects (variances)	
IIV_CL _ped_	0.529 ± 0.232
IIV_CL_adult_	0.158 ± 0.0312
IIV_*V_C_*_ped_	0.235 ± 0.0651
IIV_*V_C_*_adult_	0.132 ± 0.0436
IIV_*Q*	0.690 ± 0.165
IIV_*V_P_*	0.295 ± 0.0865
IIV_*K_a_*	0.201 ± 0.0572
Residual error random effects (variances)	
Capsule, pediatric studies	0.462 ± 0.0287
Solution, pediatric studies	0.783 ± 0.101
Adult study	0.212 ± 0.00863

a Pediatric (ped) and adult reference (ref) values are given for CL, *V_C_*, and *K_a_*. Other values for these parameters are given with respect to the reference values. IIV, interindividual variability.

### (ii) High variability and overlapping range of CL values in each genotype group.

**FIG 4:**
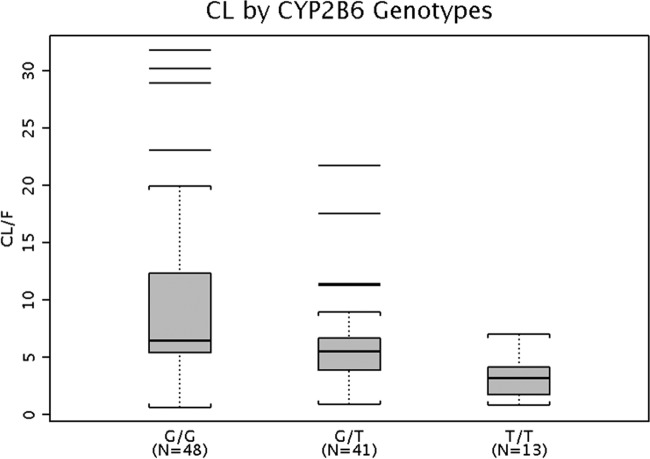
EFV apparent oral clearance (CL) by CYP2B6 genotype. Median, center line within box; 25th and 75th percentiles, lower and upper box boundaries, respectively; 90% confidence intervals, whiskers; outliers, black horizontal lines.

Assessing the CYP2B6 polymorphism as a covariate in the final EFV population PK model effect identified the population mean difference of EFV CL for subjects with different CYP2B6 genotypes. However, given the interindividual variability in CL, this estimated difference may not be apparent for a given individual due to the wide overlap in range of estimated CL values for each genotype. Figure 4 shows the *post hoc* individual pediatric subjects' EFV CL predictions grouped by their known CYP2B6 genotypes. There is high variability in EFV CL values within each CYP2B6 polymorphism subgroup, especially the CYP2B6 wild type, whose range overlaps the ranges of the CYP2B6 heterozygous mutation (15631GT) and homozygous mutation (15631TT) subgroups.

### (iii) Mixture model assessment.

A mixture model was developed to further explore the impact of CYP2B6 genetic polymorphisms and other potential causative covariates on EFV systemic exposure. The final mixture model identified estimates of CL in two subgroups (extensive and poor metabolizers), separately for adults and pediatrics patients, as well as the proportion of adult and pediatric patients who are extensive or poor metabolizers. The parameter estimates for the final mixture model are shown in Table 6.

**TABLE 6:** Final parameter estimates for the mixture model

Effect type and parameter^*a*^	Estimate ± SE
Fixed effects	
CL_ref,ped_ (liters/h)	5.33 ± 0.218
CL_ref,adult_ (liters/h)	4.98 ± 0.465
CL_ped,WT_	0.558 ± 0.0771
CL_ped,PART_	−0.0465 ± 0.0910
CL_rel,ped,group 2_^*b*^	0.831 ± 0.113
CL_rel,adult,group 2_^*b*^	0.504 ± 0.0446
*V_C_*_ref,ped_ (liters)	82.8 ± 9.53
*V_C_*_ref, adult_ (liters)	187 ± 15.1
*V_C_*_ped,WT_	1.34 ± 0.188
*Q*_ref_ (liters/h)	5.69 ± 0.845
*V_P_*_ref_ (liters)	289 ± 35.2
*K_a_*_ref_ (h^−1^)	0.423 ± 0.0515
*K_a_*_ped,WT_	0.737 ± 0.156
*T*_lag_ (h)	0.629 ± 0.0393
*F*_rel_ for solution	−0.385 ± 0.0712
Study PACTG 382	
Study PACTG 1021	−0.329 ± 0.0707
Study AI266922	−0.750 ± 0.0519
*P*_ref,ped,group 1_	0.529 ± 0.0851
*P*_ref,adult,group 1_	0.552 ± 0.135
IIV random effects (variances)	
IIV_CL_ped_	0.0357
IIV_CL_adult_	0.0538
IIV_*V_C_*_ped_	0.238
IIV_*V_C_*_adult_	0.130
IIV_*Q*	0.826
IIV_*V_P_*	0.294
IIV_*K_a_*	0.195
IIV_ CL_rel,ped,group 2_^*b*^	0.788
IIV_ CL_rel,adult,group 2_^*b*^	0.0288
Residual error random effects (variances)	
Capsule, pediatric studies	0.453 ± 0.0273
Solution, pediatric studies	0.717 ± 0.0815
Adult study	0.212 ± 0.00863

a Pediatric (ped) and adult reference (ref) values are given for CL, *V_C_*, and *K_a_*. Other values for these parameters are given with respect to the reference values. IIV, interindividual variability.

b Values are relative (rel) to those for group 1.

**FIG 5:**
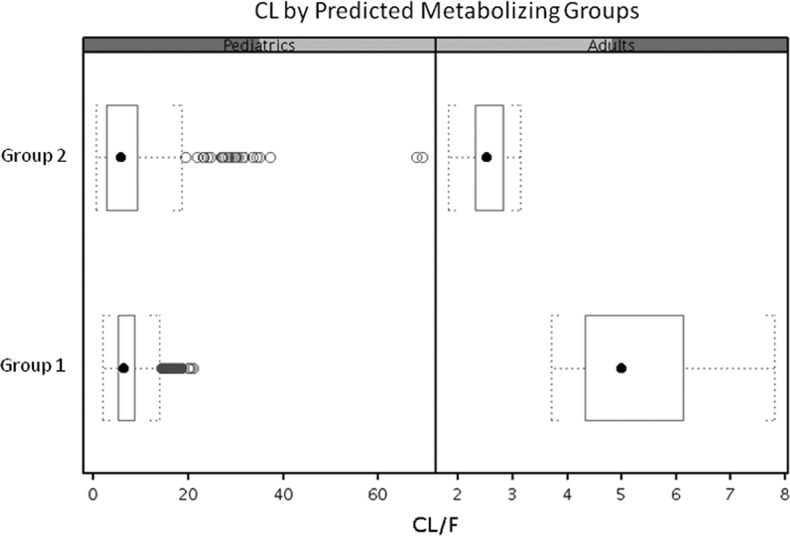
EFV CL by predicted metabolizing groups in pediatric and adult patients. Median, center line within box; 25th and 75th percentiles, lower and upper box boundaries, respectively; 90% confidence intervals, whiskers; outliers, black open dots.

The model predicted two EFV CL populations (group 1 and group 2) in both children and adults (Fig. 5). The estimated CLs of group 2 were approximately 51% lower for adult subjects and 17% lower for pediatric patients than the respective values for group 1 (Table 5).

## DISCUSSION

Population pharmacokinetic (PPK) analysis is now widely accepted as the approach to determine doses that achieve target exposures, and there are numerous studies confirming this approach in the development of drugs for pediatric populations (18–20). A model-based approach allows for the integration of adult and pediatric data, or of data from different pediatric age groups, to support the use of an approved drug in a new population (13, 21, 22). The model developed in the present analysis focused on characterizing the PK of EFV in pediatric patients and aimed to determine appropriate doses for use in infants and children aged <3 years or in those weighing <10 kg. The dosage guidelines developed achieve comparable exposures to those observed in children weighing ≥10 kg who received the previously approved dosage (200 mg QD) of EFV. Steady-state EFV concentration-time profiles in HIV-infected children were adequately described by a two-compartment PK model with first-order absorption and elimination. Body weight was identified as a clinically meaningful covariate for EFV CL, *V_C_*, and *K_a_*. Furthermore, prior antiretroviral therapy was a significant covariate with respect to CL; however, caution should be exercised in interpreting this effect as prior antiretroviral therapy is likely confounded with the effect of study PACTG1021 on CL. Therefore, it could be a study effect instead of the effect of prior antiretroviral therapy. Also, it is worth mentioning that PK parameters were estimated separately for pediatric patients and adult subjects in this model. These parameters capture not only the potential PK difference between different age groups (pediatric and adult) but also different disease states (HIV-infected patients and healthy subjects). Therefore, caution should be exercised in comparing parameter value estimates for these two populations.

Model-based simulation was employed to determine weight-tiered EFV doses (capsules or capsule sprinkles) that were projected to provide AUC_ss_, *C*_max_, and *C*_min_ values that were within target ranges for pediatric HIV-infected patients weighing ≥3.5 to <10 kg. The simulation results also confirmed the current dosing recommendations for subjects weighing ≥10 kg. As the target systemic exposures were achieved by dosing according to weight alone, further dose adjustments according to the other covariates identified in the final model were not deemed necessary.

The impact of pharmacogenomic polymorphisms on EFV exposure was also assessed, initially by incorporating the individual CYP2B6 genotype (with only limited data) as a covariate in the final EFV population PK model and further by the mixture model. A population modeling approach incorporating a mixture model can identify distinct subpopulations where potentially causative covariates, such as CYP2B6 SNPs, were not measured or where only limited numbers of the covariates of interests were collected. This approach can also be used to predict the effect of otherwise unknown genetic determinants of EFV CL in pediatric patients using observed pharmacokinetic characteristics in other patients with known phenotypes. This holistic approach enables the estimation of multimodal CL distribution in adults and pediatric patients using the entire population PK analysis data set rather than only the data from subjects for whom pharmacogenomic information is available. It should also be noted that the available CYP2B6 pharmacogenomic data in pediatric subjects represents an incomplete characterization of the CYP2B6 status of these patients, given the large number of associated SNPs (and haplotypes) (23). The mixture model predicted two EFV CL populations in both children and adults, with a much smaller difference for pediatric than adult subjects between the two EFV CL subpopulations. The size of the effect on EFV CL between the two populations was also found to be smaller in pediatric than adult subjects. This difference may due to the different development stages of the CYP2B6 metabolic enzyme in pediatric and adult subjects.

Previously, EFV was not recommended for children <3 years of age or in those weighing <10 kg because of limited safety and efficacy data. The three studies in pediatric patients reported positive efficacy and acceptable safety data (3). Also, the various systemic exposures of EFV found in all CYP2B6 genotypic subgroups did not appear to be associated with an increase in incidence or severity of EFV-associated adverse events (AEs), including rash and central nervous system adverse events.

The PK modeling and simulations presented herein enabled identification of appropriate dosage guidelines for children less than 3 years of age, and this has subsequently led to the approval of the drug in the United States and the European Union for pediatric patients aged 3 months or older and weighing between 3.5 and 40 kg. As with previous dosing guidelines for children, the recommended dose of EFV capsule (intact or sprinkle) is based on body weight, ranging from 100 mg QD for children weighing 3.5 kg to <5 kg to 400 mg QD for children weighing 32.5 kg to <40 kg. In children and adolescents weighing ≥40 kg, the recommended dose is 600 mg QD (3).

### Conclusions.

The EFV PK in pediatric HIV patients was best described by a two-compartment model with first-order absorption and elimination using a nonlinear mixed-effects modeling approach. Body weight was identified as a significant predictor of CL, *V_C_*, and *K_a_*, and the history of prior antiretroviral therapy (indicator of PACTG1021) was identified as a significant predictor of CL though this may have been a study effect. The model-based simulation results reported here support a QD dosing regimen for EFV capsules when EFV is given at 100, 150, or 200 mg in pediatric patients who are 3 months of age or older and weigh 3.5 to <5 kg, ≥5 to <7.5 kg, and ≥7.5 to <10 kg, respectively. The impact of CYP2B6 genetic polymorphisms on EFV PK was also further evaluated, and results showed that the identification of CYP2B6 polymorphisms was not informative to guide pediatric dosing.

## Supplementary Material

Supplemental material

## References

[1] Bristol-Myers Squibb Canada. 2012 Sustiva (efavirenz) product monograph. Bristol-Myers Squibb Canada, Montreal, Canada.

[2] European Medicines Agency. 2016 Sustiva. European Medicines Agency, London, United Kingdom http://www.ema.europa.eu/ema/index.jsp?curl=pages/medicines/human/medicines/000249/human_med_001068.jsp&mid=WC0b01ac058001d124.

[3] Bristol-Myers Squibb. 2012 Sustiva (efavirenz) package insert. Bristol-Myers Squibb, New York, NY http://packageinserts.bms.com/pi/pi_sustiva.pdf.

[4] Ward BA, Gorski JC, Jones DR, Hall SD, Flockhart DA, Desta Z 2003 The cytochrome P450 2B6 (CYP2B6) is the main catalyst of efavirenz primary and secondary metabolism: implication for HIV/AIDS therapy and utility of efavirenz as a substrate marker of CYP2B6 catalytic activity. J Pharmacol Exp Ther 306:287–300. doi:10.1124/jpet.103.049601. 12676886

[5] Barrett JS, Joshi AS, Chai M, Ludden TM, Fiske WD, Pieniaszek HJ Jr 2002 Population pharmacokinetic meta-analysis with efavirenz. Int J Clin Pharmacol Ther 40:507–519. doi:10.5414/CPP40507. 12698988

[6] Habtewold A, Amogne W, Makonnen E, Yimer G, Riedel KD, Ueda N, Worku A, Haefeli WE, Lindquist L, Aderaye G, Burhenne J, Aklillu E 2011 Long-term effect of efavirenz autoinduction on plasma/peripheral blood mononuclear cell drug exposure and CD4 count is influenced by UGT2B7 and CYP2B6 genotypes among HIV patients. J Antimicrob Chemother 66:2350–2361. doi:10.1093/jac/dkr304. 21846671

[7] Haas DW, Ribaudo HJ, Kim RB, Tierney C, Wilkinson GR, Gulick RM, Clifford DB, Hulgan T, Marzolini C, Acosta EP 2004 Pharmacogenetics of efavirenz and central nervous system side effects: an adult AIDS clinical trials group study. AIDS 18:2391–2400. 15622315

[8] Powers V, Ward J, Gompels M 2009 CYP2B6 G516T genotyping in a UK cohort of HIV-positive patients: polymorphism frequency and influence on efavirenz discontinuation. HIV Med 10:520–523. doi:10.1111/j.1468-1293.2009.00718.x. 19486190

[9] Tsuchiya K, Gatanaga H, Tachikawa N, Teruya K, Kikuchi Y, Yoshino M, Kuwahara T, Shirasaka T, Kimura S, Oka S 2004 Homozygous CYP2B6 *6 (Q172H and K262R) correlates with high plasma efavirenz concentrations in HIV-1 patients treated with standard efavirenz-containing regimens. Biochem Biophys Res Commun 319:1322–1326. doi:10.1016/j.bbrc.2004.05.116. 15194512

[10] FDA. 2013 Sustiva (efavirenz) pediatric patients labeling update. FDA, Silver Spring, MD http://www.fda.gov/ForPatients/Illness/HIVAIDS/History/ucm350744.htm.

[11] European Medicines Agency. 2015 Assessment report: Sustiva. EMA/260283/2015. European Medicines Agency, London, United Kingdom http://www.ema.europa.eu/docs/en_GB/document_library/EPAR_-_Scientific_Discussion_-_Variation/human/000249/WC500186956.

[12] Dunne J, Rodriguez WJ, Murphy MD, Beasley BN, Burckart GJ, Filie JD, Lewis LL, Sachs HC, Sheridan PH, Starke P, Yao LP 2011 Extrapolation of adult data and other data in pediatric drug-development programs. Pediatrics 128:e1242–e1249. doi:10.1542/peds.2010-3487. 22025597

[13] Hong Y, Kowalski KG, Zhang J, Zhu L, Horga M, Bertz R, Pfister M, Roy A 2011 Model-based approach for optimization of atazanavir dose recommendations for HIV-infected pediatric patients. Antimicrob Agents Chemother 55:5746–5752. doi:10.1128/AAC.00554-11. 21930880 PMC3232801

[14] Bergstrand M, Hooker AC, Wallin JE, Karlsson MO 2011 Prediction-corrected visual predictive checks for diagnosing nonlinear mixed-effects models. AAPS J 13:143–151. doi:10.1208/s12248-011-9255-z. 21302010 PMC3085712

[15] Kaul S, Ji P, Lu M, Nguyen KL, Shangguan T, Grasela D 2010 Bioavailability in healthy adults of efavirenz capsule contents mixed with a small amount of food. Am J Health Syst Pharm 67:217–222. doi:10.2146/ajhp090327. 20101064

[16] Kowalski KG, Hutmacher MM 2001 Efficient screening of covariates in population models using Wald's approximation to the likelihood ratio test. J Pharmacokinet Pharmacodyn 28:253–275. doi:10.1023/A:1011579109640. 11468940

[17] Schwarz GE 1978 Estimating the dimension of a model. Ann Stat 6:461–464. doi:10.1214/aos/1176344136.

[18] Cella M, Gorter de Vries F, Burger D, Danhof M, Della Pasqua O 2010 A model-based approach to dose selection in early pediatric development. Clin Pharmacol Ther 87:294–302. doi:10.1038/clpt.2009.234. 20107435

[19] Jadhav PR, Zhang J, Gobburu JV 2009 Leveraging prior quantitative knowledge in guiding pediatric drug development: a case study. Pharm Stat 8:216–224. doi:10.1002/pst.394. 19610013

[20] Karlsson MO, Lutsar I, Milligan PA 2009 Population pharmacokinetic analysis of voriconazole plasma concentration data from pediatric studies. Antimicrob Agents Chemother 53:935–944. doi:10.1128/AAC.00751-08. 19075073 PMC2650527

[21] European Medicines Agency. 2006 Guideline on the role of pharmacokinetics in the development of medicinal products in the paediatric population. European Medicines Agency, London, United Kingdom http://www.ema.europa.eu/docs/en_GB/document_library/Scientific_guideline/2009/09/WC500003066.pdf.

[22] US Food and Drug Administration. 2000 Guidance for industry. E11 clinical investigation of medicinal products in the pediatric population. U.S. Food and Drug Administration, Rockville, MD http://www.fda.gov/downloads/Drugs/GuidanceComplianceRegulatoryInformation/Guidances/UCM073143.pdf.

[23] National Center for Biotechnology Information. 2014 CYP2B6 allele nomenclature. National Center for Biotechnology Information, Bethesda, MD http://www.cypalleles.ki.se/cyp2b6.htm.

[24] Starr SE, Fletcher CV, Spector SA, Yong FH, Fenton T, Brundage RC, Manion D, Ruiz N, Gersten M, Becker M, McNamara J, Mofenson LM, Purdue L, Siminski S, Graham B, Kornhauser DM, Fiske W, Vincent C, Lischner HW, Dankner WM, Flynn PM 1999 Combination therapy with efavirenz, nelfinavir, and nucleoside reverse-transcriptase inhibitors in children infected with human immunodeficiency virus type 1. Pediatric AIDS Clinical Trials Group 382 Team. N Engl J Med 341:1874–1881. 10601506 10.1056/NEJM199912163412502

[25] McKinney RE Jr, Rodman J, Hu C, Britto P, Hughes M, Smith ME, Serchuck LK, Kraimer J, Ortiz AA, Flynn P, Yogev R, Spector S, Draper L, Tran P, Scites M, Dickover R, Weinberg A, Cunningham C, Abrams E, Blum MR, Chittick GE, Reynolds L, Rathore M 2007 Long-term safety and efficacy of a once-daily regimen of emtricitabine, didanosine, and efavirenz in HIV-infected, therapy-naive children and adolescents: Pediatric AIDS Clinical Trials Group Protocol P1021. Pediatrics 120:e416–e423. doi:10.1542/peds.2006-0925. 17646352

