# Supplementary material for: Population Pharmacokinetics Analysis To Inform Efavirenz Dosing Recommendations in Pediatric HIV Patients Aged 3 Months to 3 Years
Source: Antimicrob Agents Chemother. 2016 May 23;60(6):3676–86. doi: 10.1128/AAC.02678-15 (PMC4879370; doi:10.1128/AAC.02678-15)
Supplement: Supplemental material [file supp_60_6_3676__index.html]

Population Pharmacokinetics Analysis To Inform Efavirenz Dosing Recommendations in Pediatric HIV Patients Aged 3 Months to 3 Years — Supplemental material 

# Population Pharmacokinetics Analysis To Inform Efavirenz Dosing Recommendations in Pediatric HIV Patients Aged 3 Months to 3 Years

## Supplemental material

- Supplemental file 1 -

  Table S1: parameter estimates of the final model based on model development dataset.

  PDF, 63K
