# Supplementary material for: Population Pharmacokinetics Analysis To Inform Efavirenz Dosing Recommendations in Pediatric HIV Patients Aged 3 Months to 3 Years
Source: Antimicrob Agents Chemother. 2016 May 23;60(6):3676–86. doi: 10.1128/AAC.02678-15 (PMC4879370; doi:10.1128/AAC.02678-15)
Supplement: Supplemental material [file AAC.02678-15_zac006165258so1.pdf]

TABLE S1. Parameter estimates of the final model based on model development dataset

Parameter [units]	Estimate ± standard error
Fixed Effects	
$CL_{ref, ped}$ [L/h]	4.85 ± 0.347
$CL_{ref, adult}$ [L/h]	3.66 ± 0.297
$CL_{ped, WT}$	0.619 ± 0.114
$CL_{ped, Age}$	-
$CL_{ped, Sex}$	-
$CL_{ped, Race-AA}$	-
$CL_{ped, Race-OTH}$	-
$CL_{ped, PINT}$	-
$CL_{ped, PART}$	-0.315 ± 0.111
$V_{C\ ref, ped}$ [L]	91.6 ± 9.2
$V_{C\ ref, adult}$ [L]	186 ± 15.1
$V_{C\ ped, WT}$	1.41 ± 0.164
Q_{ref} [L/h]	5.44 ± 0.736
$V_{P\ ref}$ [L]	286 ± 33.5
Ka_{ref} [h^{-1}]	0.444 ± 0.0421
$Ka_{ped, WT}$	0.653 ± 0.0966
$Ka_{ped, Age}$	-
T_{lag} [h]	0.619 ± 0.0378
Relative F_1 for solution – Study PACTG 382	−0.339 ± 0.0857
Relative F_1 for solution – Study PACTG 1021	−0.49 ± 0.0893
Relative F_1 for solution – Study AI266922	−0.756 ± 0.0493
Inter-individual (IIV) random effects	
IIV_$CL_{, ped}$	0.383 ± 0.0667
IIV_$CL_{, adult}$	0.158 ± 0.0312
IIV_$V_{C, ped}$	0.249 ± 0.0619
IIV_$V_{C, adult}$	0.131 ± 0.0454
IIV_Q	0.820 ± 0.170
IIV_V_P	0.298 ± 0.0876
IIV_Ka	0.175 ± 0.0553
Residual error random effects	
Capsule – pediatric studies	0.433 ± 0.0288
Solution – pediatric studies	0.662 ± 0.063
Adult	0.212 ± 0.00864

WT, weight; PINT, concomitant protease inhibitor; PART, prior antiretroviral therapy; T_{lag}, absorption lag time.
